# Review of the British Thoracic Society Winter Meeting 2024

**DOI:** 10.1136/bmjresp-2025-003167

**Published:** 2025-09-14

**Authors:** Anthony W Martinelli, Chloe Hughes, Matthew Steward, Max Thomas, Steven P Walker, James D Chalmers

**Affiliations:** 1 Department of Medicine, University of Cambridge, Cambridge, UK; 2 Department of Respiratory Medicine, Cambridge University Hospitals NHS Foundation Trust, Cambridge, UK; 3 Division of Respiratory Medicine and Gastroenterology, Ninewells Hospital and Medical School, Dundee, UK; 4 Academic Department of Respiratory Medicine, Royal Devon University Healthcare NHS Foundation Trust, Exeter, UK; 5 Department of Biosciences, Medical Research Council Centre for Medical Mycology, Exeter, England, UK; 6 Department of Respiratory Medicine, University Hospitals Sussex NHS Foundation Trust, Worthing, England, UK; 7 Academic Respiratory Unit, University of Bristol, Bristol, UK

**Keywords:** Pulmonary eosinophilia, Bronchiectasis, Asthma Guidelines, Lung Physiology, Pleural Disease

## Introduction

**Figure 1:**
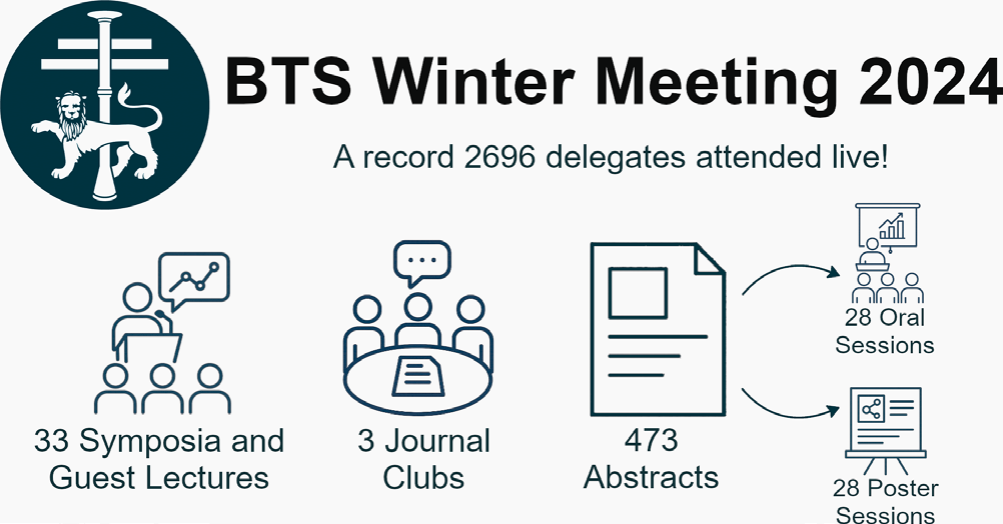
The British Thoracic Society (BTS) Winter Meeting 2024.

 It is a truth universally acknowledged that a respiratory research conference themed around literary puns is a respiratory research conference in want of a review article.^1^ Here, early career researchers (ECRs) from basic science, clinical medicine and physiology backgrounds present their personal highlights of the 2024 British Thoracic Society (BTS) Winter Meeting, held from 27 to 29 November 2024 in London and attended by a record 2696 respiratory professionals from around the world (figure 1, table 1).

**Table 1:** Demographics of attendees to the British Thoracic Society Winter Meeting 2024

Attendee profession	Number (%)
Medical	
Consultant	915 (33.9%)
General practitioner	10 (0.4%)
Associate specialist	11 (0.4%)
Specialty doctor	49 (1.8%)
Doctor in postgraduate training	444 (16.5%)
Clinical fellow	89 (3.3%)
Medical student	33 (1.2%)
Nurse	140 (5.2%)
Research scientist	106 (3.9%)
Allied health professionals	
Physiotherapist	65 (2.4%)
Speech and language therapist	11 (0.4%)
Dietitian	2 (0.1%)
Advanced clinical practitioner	18 (0.7%)
Pharmacist	34 (1.3%)
Physiologist/clinical scientist	27 (1.0%)
Physician associate	11 (0.4%)
Manager	26 (1.0%)
Industry	237 (8.8%)
Retired	10 (0.4%)
Other/unknown	459 (17.0%)
Total	2696

## Nobel laureate addresses BTS

**Figure 2:**
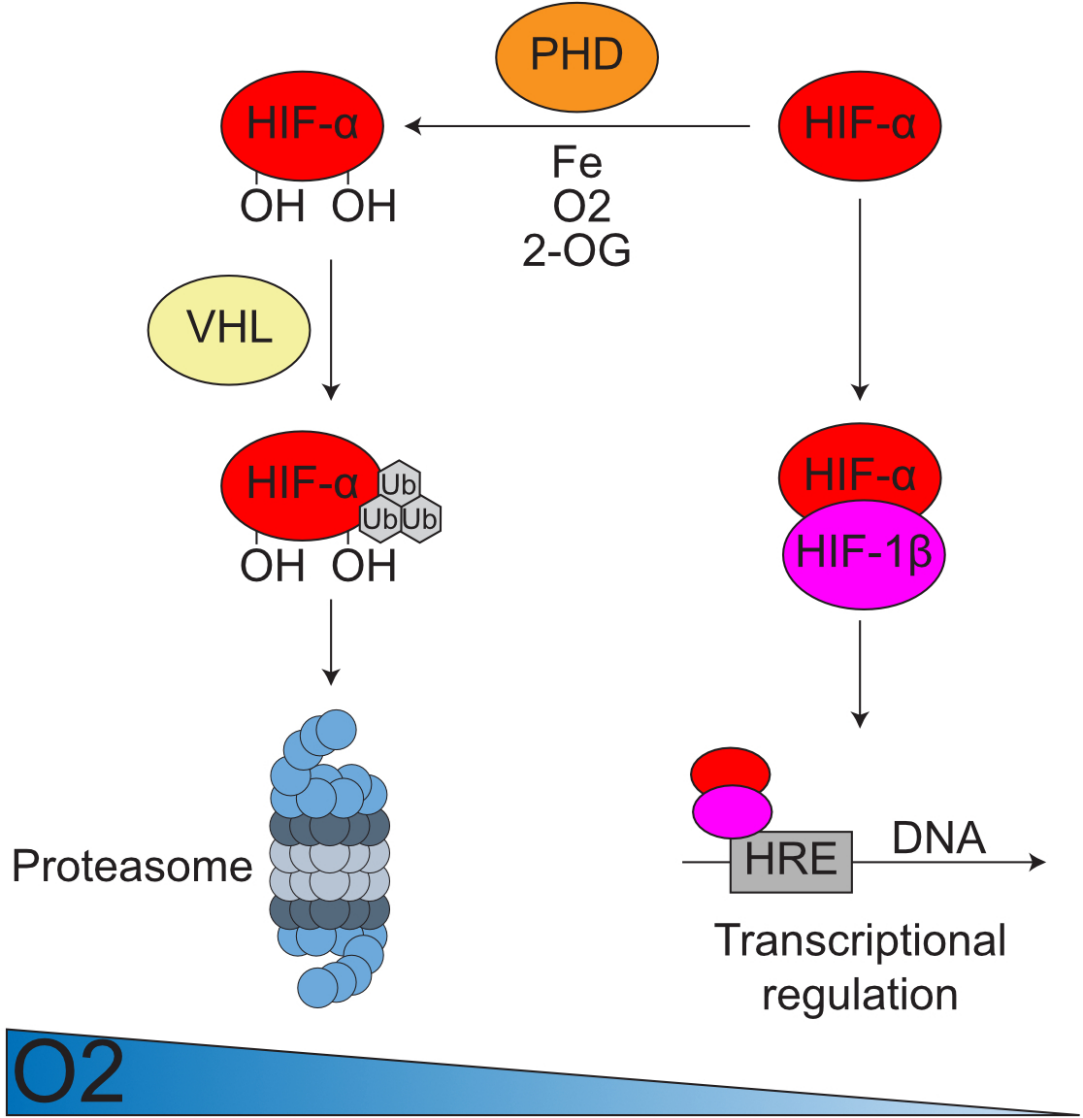
The hypoxia response. In conditions of low oxygen tension, hypoxia-inducible factor (HIF) subunits exert a transcriptional response via hypoxia response elements (HREs). When oxygen is replete, HIF-α subunits are hydroxylated by the prolyl hydroxylase domain (PHD) enzymes, which are dependent on iron and 2-oxoglutarate. They are ubiquitinated and targeted for proteasomal degradation by Von Hippel Lindau (VHL). Reproduced from thesis, A. W. Martinelli (2023)^46^ ; permission granted by the author.

Professor Sir Peter Ratcliffe (Oxford) presented the BTS Scientific Lecture on his Nobel Prize-winning work elucidating the mechanisms underlying the hypoxia response (figure 2).^2^,^4^ Further, he shared exciting new data relating to a parallel oxygen-sensing pathway, previously established in plants, which his group has found is orthologous in humans working through G protein signalling.^5^,^7^ For aspiring researchers in the audience, he provided fascinating insights into his own research career, encouraging ECRs to “find their own question” and “be brave”, as well as the more practical tip “make your own antibodies”. By pursuing research in an uncharted area, he experienced frequent setbacks and frustrating editorial decisions but reminded us that this may in fact be a positive indicator that you are researching something of truly novel biological interest. A key take-home message was that even if the significance of your work may be unclear early in the process, the wider implications of novel scientific understanding can be hugely impactful in surprising ways.

## Recognition for leading respiratory researchers

This year’s Plenary Scientific Symposium and mid-career award lectures highlighted six speakers whose impactful research careers have already changed our understanding of respiratory disease.

Professor Jennifer Quint (Imperial) took us on a journey through data science with insights from the Darzi report, highlighting the shift in focus within the National Health Service (NHS) from hospital to community, from cure to prevention and from analogue to digital. Reporting of health data is heterogeneous, with varying definitions of disease and poor clinical coding conferring huge variability to prevalence data.^8^ Professor Quint gave a compelling argument for ECRs to consider foraying into data science as a research career and reminded us that what we write in the notes has a huge impact on coding—writing ‘possible’ or ‘suspected’ is a hugely common barrier to effective coding, and something we can easily remediate to improve reporting of respiratory conditions in the UK.

Professor Karl Staples (Southampton) then gave us an insight into the mechanisms of bacterial persistence in airways disease through his recent work on non-typeable *Haemophilus influenzae* (NTHi) which are over-represented in the airways of patients with chronic respiratory disease and positively correlated with levels of neutrophilic inflammation and mortality. NTHi load can be seen to increase in hypoxia in both air–liquid interface culture and lung explants, which may be highly relevant to the changes in local oxygen availability which occur in respiratory infection. NTHi was also shown to persist in the phagosome of macrophages, including those retrieved from severe asthmatics, contributing to the chronic inflammation seen.^9^ Echoing other sessions throughout the course of this year’s meeting, Professor Staples reminded us of the forgotten impacts of inhaled and oral corticosteroids, whose overuse may be providing an ecological niche in which these pathogens can thrive.

Next up, Professor Kevin Blyth (Glasgow) gave us a unique perspective on mesothelioma research in the UK. As an ongoing global epidemic with persistently poor mortality data, the need for early diagnosis, prediction and risk profiling of patients was highlighted as a key focus in the field.^10^ With an impressive body of work spanning targeting early intervention, the use of multi-omics on longitudinal tissue samples from the Meso-ORIGINS study to engineer cell lines to undertake high-throughput drug discovery work, and ending with a tantalising look at the future with immunopeptide vaccinations, Professor Blyth gave a scintillating overview of the field.^11^

Professor Philip Molyneaux (Imperial) presented his work on the microbiome in interstitial lung disease, starting with data demonstrating high bacterial loads in patients with idiopathic pulmonary fibrosis (IPF) correlating with increased mortality.^12^ Interestingly, fibrotic hypersensitivity pneumonitis and IPF have distinct bacterial clusters, implying that there is a disease-specific factor driving this beyond the lung architectural changes relating to fibrosis. Beyond changes in bacterial burden, Professor Molyneaux also showed the inter-relationship between bacteria and short-chain fatty acids (SCFAs), with SCFA levels increasing in a dose-dependent fashion with bacterial burden, and propionate (a key SCFA) having direct deleterious effects on wound healing in air liquid interface cultures of lung fibrosis and resulting in fibroblast proliferation. IPF has limited treatment options, and so Professor Molyneaux speculated that infection may represent a novel treatment target, or biomarker, for IPF.

The BTS/Asthma + Lung UK/British Association for Lung Research (BALR) mid-career lecture awards represent the very best in UK respiratory science. This year’s BTS/ALUK winner, Dr Elaine Soon (Cambridge), presented her research elucidating dysregulated cytokine pathways which contribute to the development of pulmonary hypertension (PH).^13^ PH has a close relationship with cytokine biology, as highlighted by its high incidence in conditions with high systemic levels of inflammation such as HIV or systemic sclerosis. In Dr Soon’s murine models, this has been recapitulated by inducing systemic inflammation with lipopolysaccharide or mitomycin-c in mice with genetic knockouts of interest—in particular, the general control non-derepressible 2 gene which results in murine pulmonary veno-occlusive disease (PVOD), a particularly severe PH phenotype. Interestingly, interleukin (IL)-6 knockout mice were protected from developing PVOD in this setting, implying potential therapeutic benefits of cytokine abrogating therapies in the future for PH subtypes. This relationship with IL-6 was also demonstrated by significantly poorer survival outcomes for patients with high IL-6 levels with heritable pulmonary arterial hypertension (PAH) caused by BMPR2 mutation, the most common gene defect to cause PAH, hinting at potential future use as a prognostic biomarker.^14^

Dr Aran Singanayagam (Imperial) presented work that took a reverse translational approach, from human observation back to explorative mechanistic in vitro and in vivo studies. Interferons (IFNs) were the flavour of the day here, with fascinating data from human *Rhinovirus* challenge models showing dysfunctional IFN signalling results in viral persistence and airway inflammation. Macrophages derived from BAL of obese and non-obese patients also showed differential production of IFNs, with subsequent metabolomics elucidating dysfunctional fatty acid metabolism and AMP production.^15^ This directly affected IFN signalling, while leptin (overexpressed in obesity) promoted a proinflammatory cytokine milieu. Dr Singanayagam also demonstrated that leptin upregulates expression of suppressor of cytokine signalling-3, reducing subsequent IFN production and therefore impairing the antiviral response.^15^ Finally, IFNs and their interplay with inhaled corticosteroid (ICS) use were examined, with ICS impairing type 1 IFN production both in vitro and in murine work, with consequently enhanced viral replication.^16^ Exogenous IFN was also shown to reverse the upregulated MUC5AC production and increased bacterial load seen in the presence of ICS.

## Focus on the next generation

With Professor Nick Maskell’s Presidential address highlighting the need for a renewed focus on clinical academic training, it was heartening to see a range of presentations from developing researchers covering topics from mechanistic biology to practice-changing clinical trials.

The Early Career Investigator session aimed to showcase the best abstracts submitted to the Winter Meeting, typically as the culmination of doctoral study by basic and clinician-scientists. Dr Emma Johnson (BALR Award winner; Dundee) described her research into the mechanism of action of the dipeptidyl peptidase-1 inhibitor brensocatib, identifying that trial patients treated with the drug had widespread changes in their neutrophil proteome suggestive of a broad anti-inflammatory impact.^17^ Dr Imran Howell (Asthma + Lung UK Award winner; Oxford) presented his work from the BOOST study, which found that measuring FeNO in suspecting asthma exacerbations in patients receiving anti-IL5/IL-5R therapy could identify patients more or less likely to benefit from additional corticosteroids.^18^ Dr Hannah Schiff (BTS Award winner; Southampton) highlighted the difficulties with delayed diagnosis of tuberculosis (TB) and how investigating the plasma proteome of patients with pulmonary TB has allowed her to develop a protein panel which could help screen those at risk of the disease.^19^ Highly commended talks were given by Dr Niki Veale (Cambridge), who has developed a single-cell atlas of pleural mesothelioma, Dr Tereza Masonou (UCL), analysing the differential function of neutrophils in elderly patients in response to SARS-CoV-2, and Dr Kayesha Coley (Leicester), presenting on the genetics of chronic cough.^20^,^22^

Meanwhile, Dr Lance Burn (Cambridge), winner of the best medical student abstract award, presented his work on the use of CT features to predict recurrence of spontaneous pneumothorax, showing that the presence of contralateral lung cysts is associated with a higher risk of contralateral recurrence.^23^ Highly commended was Dr Jody Cheng (Imperial), who has developed a co-culture model of nasal epithelium and peripheral blood mononuclear cells for an in vitro study of mucosal influenza vaccines.^24^

Finally, the Specialty Trainees Advisory Group Scientific Symposium “Biography of a Lung” featured ECRs telling the story of the organ from cradle to grave. Dr Amanda Goodwin (Nottingham) commenced the session with her work on the role of the G protein α subunits Gαq and Gα11 on lung development and repair, highlighting how imbalances in this signalling pathway may drive bronchopulmonary dysplasia, emphysema and lung fibrosis.^25^ Paediatrician Dr Sormeh Salehian (Imperial) provided her perspective on how childhood exposures may influence respiratory health later in life and how a machine learning approach could help us better understand and predict this.^26^ Moving firmly into adulthood, physiologist Dr Owen Tomlinson (Exeter) championed the role of cardiopulmonary exercise testing as a valid and reliable biomarker in diseases, including interstitial lung disease and cystic fibrosis, where it may help track disease progression and prognosis.^27 28^ Lastly, Dr Wezi Sendama (Newcastle) shared his approach to tackling the challenges of an ageing immune system characterised by impaired efferocytosis, using in silico screening of connectivity maps to identify compounds which could modulate inflammation and help optimise the host response to lung infection.^29^

## Paradigm shifts in respiratory disease

The Winter Meeting made international headlines as authors presented results from landmark studies across the spectrum of respiratory disease. Top of the bill was the ABRA trial, published alongside the conference and presented by Mona Bafadhel (King’s), which investigated the use of benralizumab as a treatment for eosinophilic patients with acute exacerbations of asthma or chronic obstructive pulmonary disease (COPD).^30^ In this randomised placebo-controlled trial, both treatment failure at 90 days and visual analogue scale symptoms at 28 days were improved in patients who had received treatment with the monoclonal antibody. The encouraging results from this phase II trial will now be tested in larger studies.

**Figure 3:**
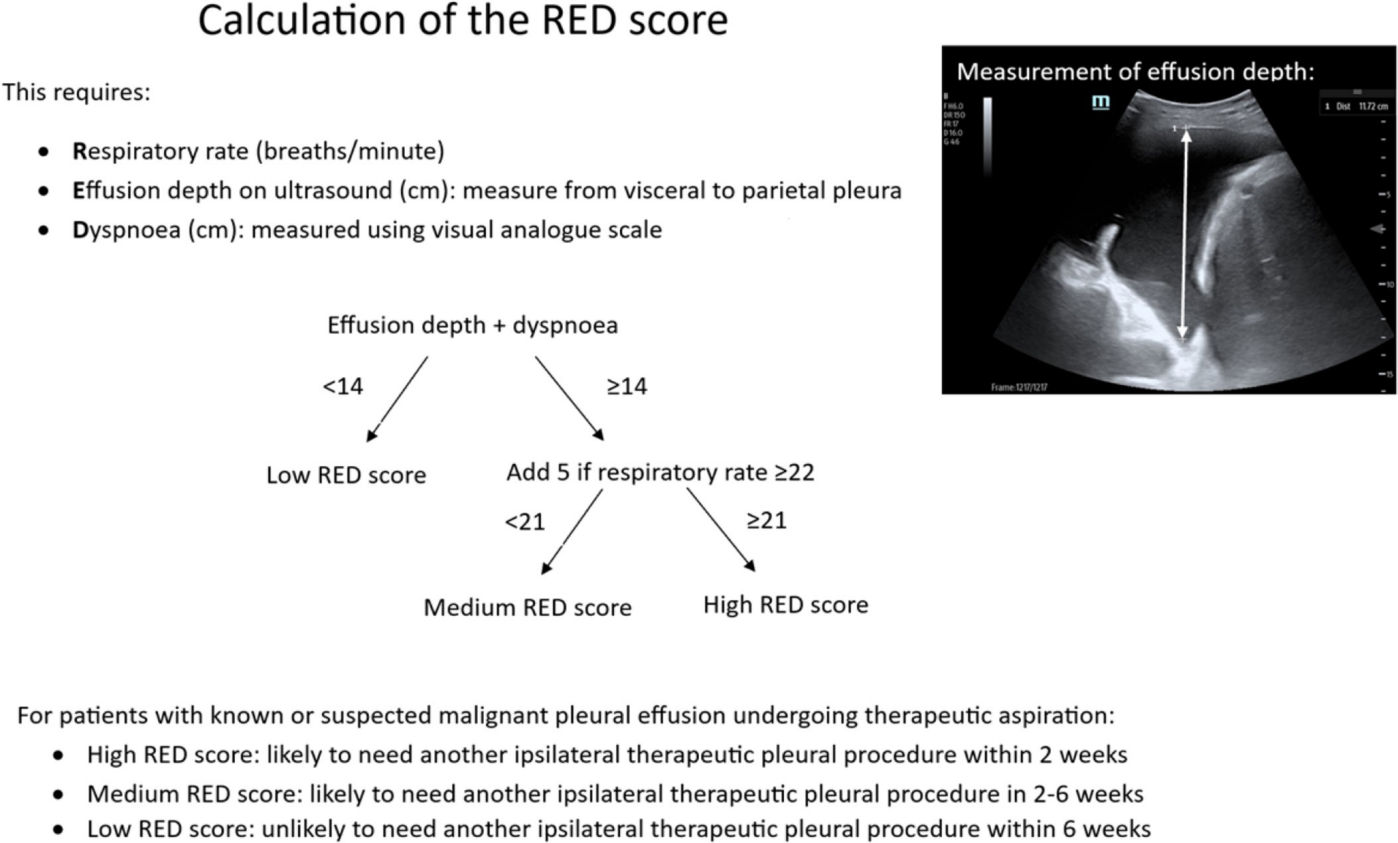
Calculation of the Respiratory rate, Effusion depth, Dyspnoea (RED) score. The RED score can be used to assess the risk of need for recurrent pleural intervention. Figure provided by Professor Eleanor Mishra and permission granted for use.

It is tough to make predictions, especially about the future. The REPEAT study sought to predict time to next pleural procedure for patients with malignant pleural disease, identifying patients at need of either early review or prompt definitive fluid control.^31^ Professor Eleanor Mishra’s (Norwich) team developed a risk prediction score for subsequent pleural procedure for patients post initial aspiration. Of the 211 patients studied, a third had their next procedure within the first 2 weeks, with another third not for the next 3 months. The team created and validated the RED score, which used three variables (respiratory rate, depth of pleural effusion on ultrasound and dyspnoea, measured on visual analogue score) (figure 3). High scores were most likely to need another procedure in 2 weeks and twice as likely to be admitted as an emergency due to breathlessness.

We were also led into a new era for bronchiectasis treatment in a symposium focused on airway clearance and anti-inflammatory therapies. Dr Arietta Spinou (King’s) presented on airway clearance techniques in bronchiectasis using data derived from EMBARC, the European Bronchiectasis Registry of over 16 000 patients from 28 countries.^32^ 72% of patients were experiencing daily expectoration of sputum and found that the active cycle of breathing technique was the most frequently used airway clearance technique. Dr Spinou further discussed the barriers to airway clearance techniques for bronchiectasis patients, which included the lack of access to a physiotherapist. Professor Felix Ringhausen (Hanover) reviewed the importance of mucociliary clearance and the pathophysiology of mucus dysfunction in bronchiectasis, with particular reference to mucus plugging. He provided an overview of the pathophysiological feed-forward cycle of mucus dysfunction which involved a loop of mucus plugging, hypoxia, increased MUC5B, increased sodium channels and mucus hypersecretion. One exciting new clinical trial aimed at translating this knowledge into clinical practice is the CLEAR trial, which is testing hypertonic saline and carbocisteine for airway clearance compared with usual care.^33^ Professor James Chalmers (Dundee) presented results from the phase III ASPEN trial of brensocatib, which, when given over a 52-week treatment period, reduced the frequency of exacerbations, prolonged time to first exacerbation and reduced lung function decline compared with placebo.^34^

**Figure 4:**
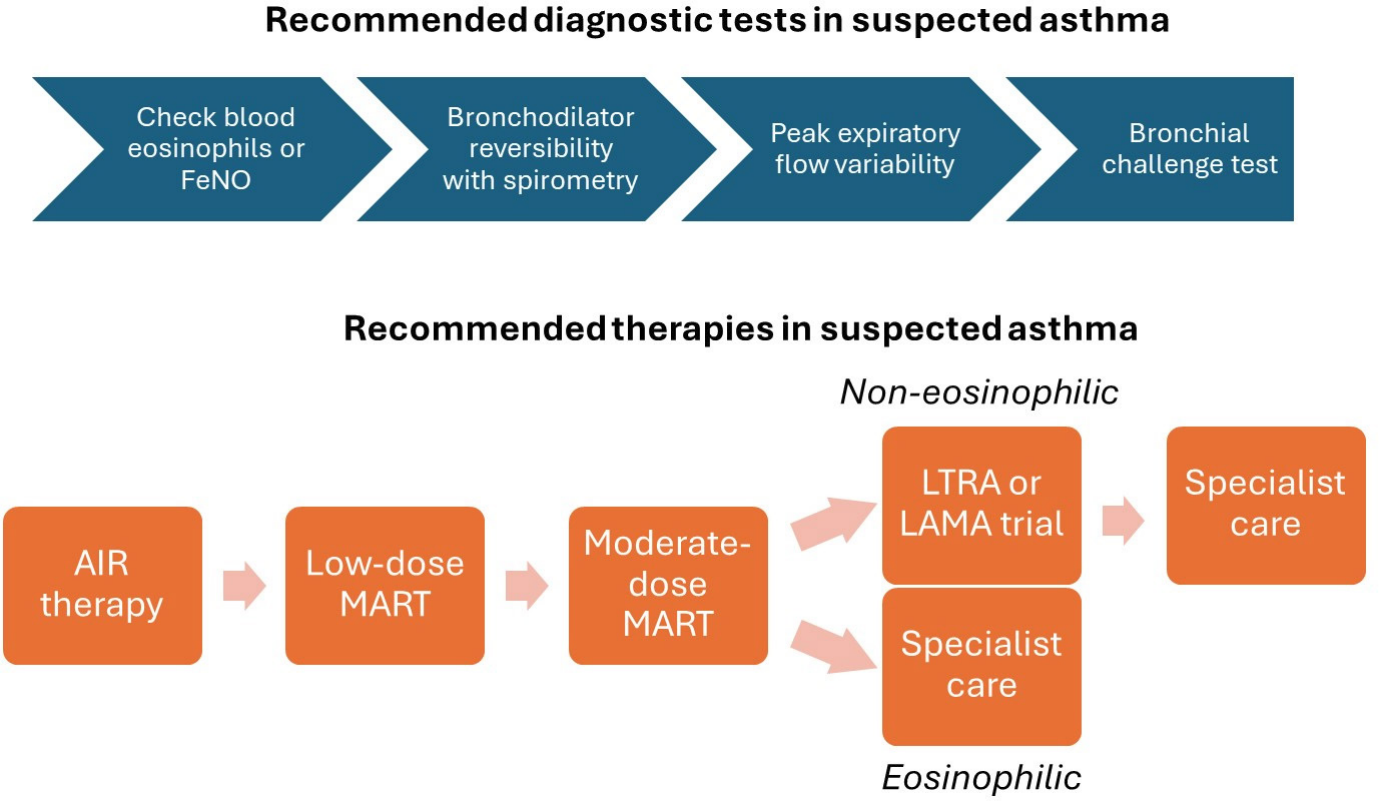
New diagnostic and therapeutic algorithms for chronic asthma management in adults. Algorithms adapted from the National Institute for Health and Care Excellence guideline 245. Sequential diagnostic tests are recommended in patients with a history suggestive of asthma. Treatments suggested to be escalated iteratively depending on response. AIR, anti-inflammatory reliever; FeNO, fractional exhaled nitric oxide; LAMA, long-acting muscarinic antagonist; LTRA, leukotriene receptor antagonist; MART, maintenance and reliever therapy.

Finally, it would not be the BTS Winter Meeting without the publication of a major new guideline. This year, it was the turn of the asthma community to revel in the excitement and controversy that surrounds the release of such documents, in this case co-produced for the first time by BTS, the Scottish Intercollegiate Guidelines Network and the National Institute for Health and Care Excellence. Among the major changes made to the recommended management of chronic asthma was a much-reduced role for the short-acting beta agonist ‘blue inhaler’, to be replaced with an anti-inflammatory reliever consisting of low-dose ICSs and formoterol in combination, followed by progression to Maintenance and Reliever Therapy, where symptoms are uncontrolled (figure 4). A new diagnostic algorithm was also developed for the guidance, recommending sequential measurement of blood eosinophils or FeNO, bronchodilator reversibility with spirometry, peak flow variability and bronchial challenge testing in patients with a history suggestive of asthma.^35^

## Beyond pathophysiology: social determinants of health, audit and the multiprofessional workforce

The BTS Grand Challenge Lecture was delivered by Sarah Woolnough, chief executive of The King’s Fund and erstwhile chief executive of Asthma + Lung UK, posing the question, “What would it take to improve lung health outcomes a decade from now?”. Though current issues including long waits, rushed appointments, worsening basic care in COPD and asthma, and low staff morale were fully acknowledged, an optimistic vision was presented, highlighting how by targeted action these trends could be reversed. Focuses on prevention and tackling inequalities, empowering individuals and communities, and providing the resource to deliver high-quality care were among the key potential interventions. The substantial link between deprivation and respiratory health was noted as a particular concern, with the most deprived developing long-term conditions a decade before their peers.^36^ The roles of the data revolution and partnerships with industry were also highlighted as having the potential to drive meaningful improvements for respiratory patients.^37^ Finally, a clear theme of the presentation was the need for responsive leadership across boundaries, listening to people and targeting the areas of greatest need.

**Figure 5:**
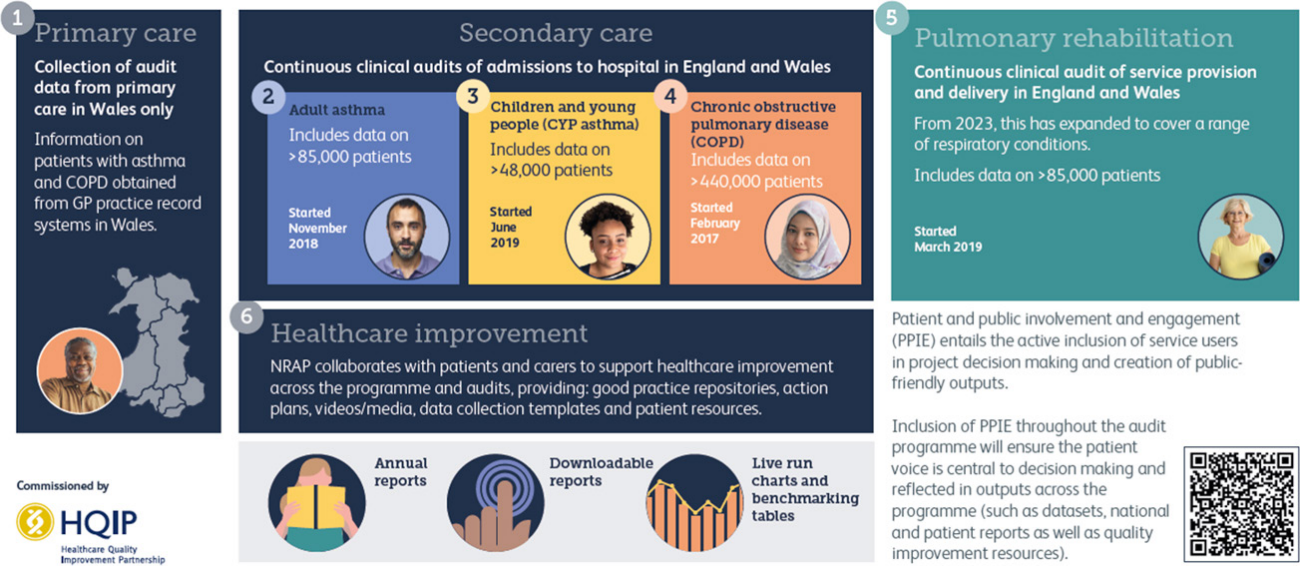
Overview of the National Respiratory Audit Programme (NRAP). Figure provided by Professor Alice Turner and permission granted for use by Professor Tom Wilkinson on behalf of the NRAP. GP, general practitioner.

Further focus on improving care was provided to delegates receiving an update on the National Respiratory Audit Programme (NRAP), led by the Royal College of Physicians. A cornerstone initiative driving improvements in respiratory care across the UK, by auditing key conditions such as asthma and COPD, NRAP gathers comprehensive data on clinical outcomes and adherence to best practice guidelines (figure 5).^38^ This evidence-based approach allows healthcare providers to benchmark performance, identify disparities and implement targeted quality improvements. NRAP stands out as a powerful tool for advancing respiratory care, fostering accountability and enabling multidisciplinary teams to deliver higher standards of treatment for patients nationwide. These data now encompass over 500 000 patients with COPD and asthma, and make patient and public involvement and engagement a core part of the project.

Professor Alice Turner (Birmingham) presented the NRAP Healthcare Improvement (HI) Training Programme, which supports teams undertaking healthcare improvement projects.^39^ The programme includes online education delivered by leaders in healthcare improvement, followed by action learning techniques facilitated by experienced coaches. This approach has already yielded success, with 30 teams implementing projects to enhance quality in their healthcare systems. For example, the John Radcliffe Hospital increased smoking cessation referrals for COPD admissions to 90.3%, while Barnsley’s pulmonary rehabilitation service achieved 100% of patients being seen within 90 days of referral. The HI programme is open to all healthcare professionals, and the organisers are particularly keen to hear from physiologists interested in participating next year. The application timeline for teams is expected to remain similar to this year. Notably, there is likely to be an increased focus on primary care in England in the coming years, opening opportunities for work on diagnostics. Addressing gaps such as the persistently low proportion of COPD admissions with spirometry-confirmed diagnoses remains a priority—an area where physiologists could play a critical role, including through secondary care audits. Find out more at https://www.rcp.ac.uk/improving-care/national-clinical-audits/the-national-respiratory-audit-programme.

Workforce pressures were a key theme of many of the Specialist Advisory Group (SAG) open meetings taking place across the Winter Meeting. For example, at the joint Association for Respiratory Technology & Physiology (ARTP)/BTS SAG meeting, Dr Joanna Shakespeare and Dr Martin Allen highlighted the pressing challenges facing the respiratory physiology workforce, particularly the lack of trainees entering undergraduate-level practitioner training programmes. He emphasised that the insufficient investment in workforce training is increasingly undermining the ability of services to deliver high-quality care across the UK. The urgent need to prioritise and fund workforce development was underscored as a critical component of maintaining and improving respiratory services nationally. There were, however, many recent reasons for optimism about the future, including securing a contract with *BMJ Open Respiratory Research* to publish ARTP conference abstracts, collaborating with chief scientific officers from the four nations to advance respiratory science, engaging with Higher Education Institutions to discuss professional qualifications and launching the first ARTP research course.

Finally, while medical doctors remained the most well-represented attendees at the conference (table 1), promoting the scientific contributions of the wider multiprofessional team remains a priority for the BTS Winter Meeting. The Evidence-Informed Nurse Led Practice symposium included Vicki Slade, Sarah Kearney and Professor Lynn Calman celebrating the contributions of nurses to care outside of hospital and in leading research. In the same symposium, Sally Bustin was joined by physiotherapist-scientist Dr Sara Buttery to give an overview of the role of the nurse in lung volume reduction surgery, an area of speaker expertise.^40^

## Reflections on the past and visions for the future

Completing the showcase sessions was Professor Stuart Elborn (Belfast), recipient of the BTS Medal for his contribution to the care of patients with cystic fibrosis, who delivered the BTS Clinical Lecture on ‘Academic Medicine: Trials and Tribulations’. Reflecting on four decades of clinical practice and leading research, he took the audience on a journey from Kierkegaard (a personal inspiration) to the challenges of treating patients with multimorbidity.^41^,^43^ In addition to making a powerful case for fairer funding for respiratory research, he focused on the need for well-designed clinical trials run by effective teams to maximise patient benefit. Looking forward, he highlighted the potential of ‘digital twinning’ which seeks to leverage generative artificial intelligence to repurpose existing data as controls in both basic science experiments and clinical trials, streamlining the scientific process.^44^ Finally, summarising the themes of the Winter Meeting, he emphasised the importance of asking the right questions, multidisciplinary collaboration and training the next generation.^45^

## Concluding remarks

These personal highlights represent only a fraction of the work presented at the UK’s largest respiratory research conference. As time and resource available for NHS clinicians to undertake research have become increasingly scarce, the authors would like to take this opportunity to thank and congratulate all who chose to submit their work to the 2024 BTS Winter Meeting and encourage many more to consider doing so in 2025.

So we beat on, cilia against the current, borne back ceaselessly into the past.
